# Basal *hsp70* expression levels do not explain adaptive variation of the warm- and cold-climate O_3 + 4 + 7_ and O_ST_ gene arrangements of *Drosophila subobscura*

**DOI:** 10.1186/s12862-020-1584-z

**Published:** 2020-01-31

**Authors:** Marta Puig Giribets, Mauro Santos, María Pilar García Guerreiro

**Affiliations:** Grup de Genòmica, Bioinformàtica i Biologia Evolutiva (GGBE), Departament de Genètica i de Microbiologia, Universitat Autònoma de Barcelona, Bellaterra, 08193 Barcelona, Spain

**Keywords:** *Drosophila subobscura*, *hsp70* expression, Chromosomal arrangements, Thermal adaptation

## Abstract

**Background:**

*Drosophila subobscura* exhibits a rich inversion polymorphism, with some adaptive inversions showing repeatable spatiotemporal patterns in frequencies related to temperature. Previous studies reported increased basal HSP70 protein levels in homokaryotypic strains for a warm-climate arrangement compared to a cold-climate one. These findings do not match the similar *hsp70* genomic organization between arrangements, where gene expression levels are expected to be similar. In order to test this hypothesis and understand the molecular basis for *hsp70* expression, we compared basal *hsp70* mRNA levels in males and females, and analysed the 5′ and 3′ regulatory regions of *hsp70* genes in warm- and cold-climate isochromosomal O_3 + 4 + 7_ and O_ST_ lines of *D. subobscura*.

**Results:**

We observed comparable mRNA levels between the two arrangements and a sex-biased *hsp70* gene expression. The number of heat-shock elements (HSEs) and GAGA sites on the promoters were identical amongst the O_ST_ and O_3 + 4 + 7_ lines analysed. This is also true for 3′ AU-rich elements where most A and B copies of *hsp70* have, respectively, two and one element in both arrangements. Beyond the regulatory elements, the only notable difference between both arrangements is the presence in 3′ UTR of a 14 bp additional fragment after the stop codon in the *hsp70*A copy in five O_3 + 4 + 7_ lines, which was not found in any of the six O_ST_ lines.

**Conclusions:**

The equivalent *hsp70* mRNA amounts in O_ST_ and O_3 + 4 + 7_ arrangements provide the first evidence of a parallelism between gene expression and genetic organization in *D. subobscura* lines having these arrangements. This is reinforced by the lack of important differential features in the number and structure of regulatory elements between both arrangements, despite the genetic differentiation observed when the complete 5′ and 3′ regulatory regions were considered. Therefore, the basal levels of *hsp70* mRNA cannot account, in principle, for the adaptive variation of the two arrangements studied. Consequently, further studies are necessary to understand the intricate molecular mechanisms of *hsp70* gene regulation in *D. subobscura*.

## Background

Inversions can foster local adaptation as they protect chromosomal inverted sequences from recombination in heterokaryotypes. *Drosophila subobscura* exhibits a rich inversion polymorphism [1], with the O chromosome (Muller element E that also corresponds to chromosome arm 3R in *Drosophila melanogaster* ([2], p., 307) having the highest number of inversions that form complex gene arrangements of overlapping and non-overlapping inversions [3]. The overlapping inversions cluster in the distal segment of the chromosome and form the ${\mathrm{O}}_{\underset{\_}{3+4}}$ phylad, whose different gene arrangements have higher frequencies at lower latitudes [1, 4]. Thus, the gene arrangements on chromosome O can be divided into two groups: group 1 (cold-climate) includes O_ST_ that shows a negative correlation coefficient with maximum temperatures along the latitudinal clines, or a positive correlation coefficient with latitude; and group 2 (warm-climate) that includes gene arrangements showing the reverse pattern (e.g., ${\mathrm{O}}_{\underset{\_}{3+4}}$, ${\mathrm{O}}_{\underset{\_}{3+4}+7}$ and ${\mathrm{O}}_{\underset{\_}{3+4+8}}$). In agreement with the clinal patterns, warm-climate inversions increase in frequency during summer and decrease during winter [5, 6]. Furthermore, they show long-term trends of increasing frequency related to global warming [7, 8]. Overall, these spatiotemporal patterns strongly suggest that temperature is the main selective factor driving inversion frequencies, but the underlying genetic causes are still poorly understood.

The heat-shock HSP70 protein is involved in protein folding, assembly, and can be induced by several stressors such as a sudden increase in temperature [9, 10]. It appears to be the primary protein involved in thermotolerance in *D. melanogaster* [11, 12], and allele frequencies show latitudinal clines and change in response to thermal evolution in the laboratory [13]. In a previous work with *D*. *subobscura*, expression levels of HSP70 protein in flies carrying cold- and warm-climate gene arrangements were analysed [14]. It was found that homokaryotypic ${\mathrm{O}}_{\underset{\_}{3+4}}$ flies had increased HSP70 basal protein levels that did not boost after a heat-shock, in contrast to what happened to their O_ST_ homokaryotypic counterparts. This result was somehow surprising because HSP70 protein expression entails fitness costs [15–17]. The interpretation was that low-latitude populations with relatively high ${\mathrm{O}}_{\underset{\_}{3+4}}$ frequencies are more likely to be exposed to occasionally higher temperatures than populations located at higher latitudes, and the benefits of mounting the stress response might outweigh the costs. Actually, an extreme heat wave in Europe in spring 2011 caused frequencies of warm-climate ${\mathrm{O}}_{\underset{\_}{3+4}+7}$ to shift +5.4*σ* from the expected average frequency (in close agreement with the temperature anomaly), and high selection coefficients are needed to explain this shift [18].

This interpretation is not without caveats. A trend to a decrease in basal HSP70 protein expression was observed in strains of *D. melanogaster* [19, 20] and *Drosophila buzzatii* [21] continually exposed to high temperatures, probably to avoid the deleterious effects of increased HSP70 levels. On the other hand, increased basal HSP70 protein and mRNA levels have been observed in certain strains of *Drosophila virilis* with increased thermotolerance [22]. Therefore, basal levels of HSP70 protein expression might be contingent on the thermal history and the species under study. A positive link between *hsp70* copy number, expression levels, and thermal resistance in artificial [23–25] and natural [26] *Drosophila* strains has been reported, but this does not apply to *D*. *subobscura*. In this species, we have recently shown that the genomic sequence organization of the *hsp70* gene family (located in the 94A subsection of the O chromosome, which is inside the region covered by O_3_ and O_4_ inversions [27];), is essentially similar in cold- (O_ST_) and warm-climate (${\mathrm{O}}_{\underset{\_}{3+4}}$, ${\mathrm{O}}_{\underset{\_}{3+4+8}}$ and ${\mathrm{O}}_{\underset{\_}{3+4+16}}$) gene arrangements, and consists of only two genes, each with four heat-shock elements and three GAGA sites on its promoter [27]. To date, no clear evidence has been found to link the genomic sequence organization of the *hsp70* gene family with the different HSP70 protein expression patters previously detected [14].

Knowing that proteins are produced from mRNA which, in turn, depends on gene regulatory regions, we extended the former approach to gauge if *hsp70* mRNA levels are similar in cold- and warm-climate arrangements as expected by their similar genomic organization. We quantified basal *hsp70* mRNA expression levels in males and females, and included a higher number of isogenic O_ST_ and ${\mathrm{O}}_{\underset{\_}{3+4}+7}$ strains than in our previous study [27]. We expanded the study to the 5′ and 3′ *hsp70* regulatory regions, which are known to be involved in transcriptome variation in *D. melanogaster* [28]. A clarification is in order. The arrangement ${\mathrm{O}}_{\underset{\_}{3+4}+7}$ has the overlapping inversions ${\mathrm{O}}_{\underset{\_}{3+4}}$ in the distal segment and the single O_7_ inversion in the proximal segment of chromosome O. Because our lines derived from a natural population where both gene arrangements (O_ST_ and ${\mathrm{O}}_{\underset{\_}{3+4}+7}$) have a relatively high frequency (Berbikiz, north of Spain [18]) the heterokaryotype ${\mathrm{O}}_{\underset{\_}{3+4}}/{\mathrm{O}}_{\underset{\_}{3+4}+7}$ freely recombines in the distal segment and, therefore, the gene content in the ${\mathrm{O}}_{\underset{\_}{3+4}}$ region is expected to be the same in both arrangements. The reason for using ${\mathrm{O}}_{\underset{\_}{3+4}+7}$ was because the integrity of the wild chromosome is better preserved during the isogenization process with the available balancer stock in *D*. *subobscura* (see below).

We found equivalent mRNA amounts in O_ST_ and ${\mathrm{O}}_{\underset{\_}{3+4}+7}$ arrangements, a sex-biased *hsp70* mRNA gene expression compatible with the high degree of conservation of the cis-regulatory elements observed, and an identical genomic organization of the gene family previously reported in our study.

## Results

### High degree of conservation in the *hsp70* proximal and distal CRE elements between O_ST_ and O_3 + 4 + 7_ arrangements

**Fig. 1:**
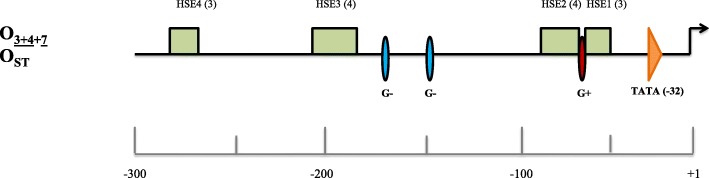
Representation of *hsp70* 5′ *cis*-regulatory elements of O_ST_ and O_3 + 4 + 7_ lines. The two copies of the gene (A and B) are included. Green rectangles: HSEs 1–4, where numbers in brackets represent the number of nGAAn or nTTCn pentanucleotide units. Red ellipse: GAGAG (G+) repeat motifs. Blue ellipses: CTCTC (G-) repeat motifs. Orange triangle: TATA box. The scale represents nucleotides relative to the transcription start site (+ 1), indicated by a forward arrow

We have sequenced the proximal and distal regulatory regions of the *hsp70* locus in 12 isogenic lines (6 lines for O_ST_ and 6 for ${\mathrm{O}}_{\underset{\_}{3+4}+7}$ gene arrangements). The proximal 5′ region consists of a ~ 550 bp, of which ~ 290 bp correspond to the region upstream of the transcription start site (TSS) in the two *hsp70* gene copies (*hsp70A* and *hsp70B*). Multiple sequence alignments indicated high CRE (Cis-Regulatory Element) conservation between O_ST_ and ${\mathrm{O}}_{\underset{\_}{3+4}+7}$. As previously found [27], four highly conserved heat shock elements (HSEs) were identified (Fig. 1), and no fixed nucleotide differences between arrangements were observed (Additional files 1 and 2). The genomic organization of HSEs resembles that of other *Drosophila* species [29] and consists of at least three continuous alternate nGAAn/nTTCn pentanucleotide subunits representing binding sites for the heat shock factor (HSF) monomer. Although the number of repetitions is identical in the 12 isogenic lines, it varies between HSE sequences: three in the most distal and proximal (HSE1, HSE4) and four in HSE2 and HSE3 (Fig. 1). Within each HSE, polymorphisms appear to be overrepresented in the first and fifth position of nGAAn/nTTCn pentanucleotides, which are the least constrained [29]. Some insertion/deletion (INDEL) polymorphisms were also detected in the presumably unconstrained regions separating the more distal HSEs. These polymorphisms tend to be shared between the two *hsp70* paralogs of the same line as a result of ectopic gene conversion [27]. We did not observe differences between isogenic lines either in the two core proximal promoter elements (TATA box and TSS), or in the total number of GAGA sites in the proximal promoters. The latter is known to be recognized by the transcription factor GAF and to participate, like other promoters, in the regulation of global gene expression [30].

**Fig. 2:**
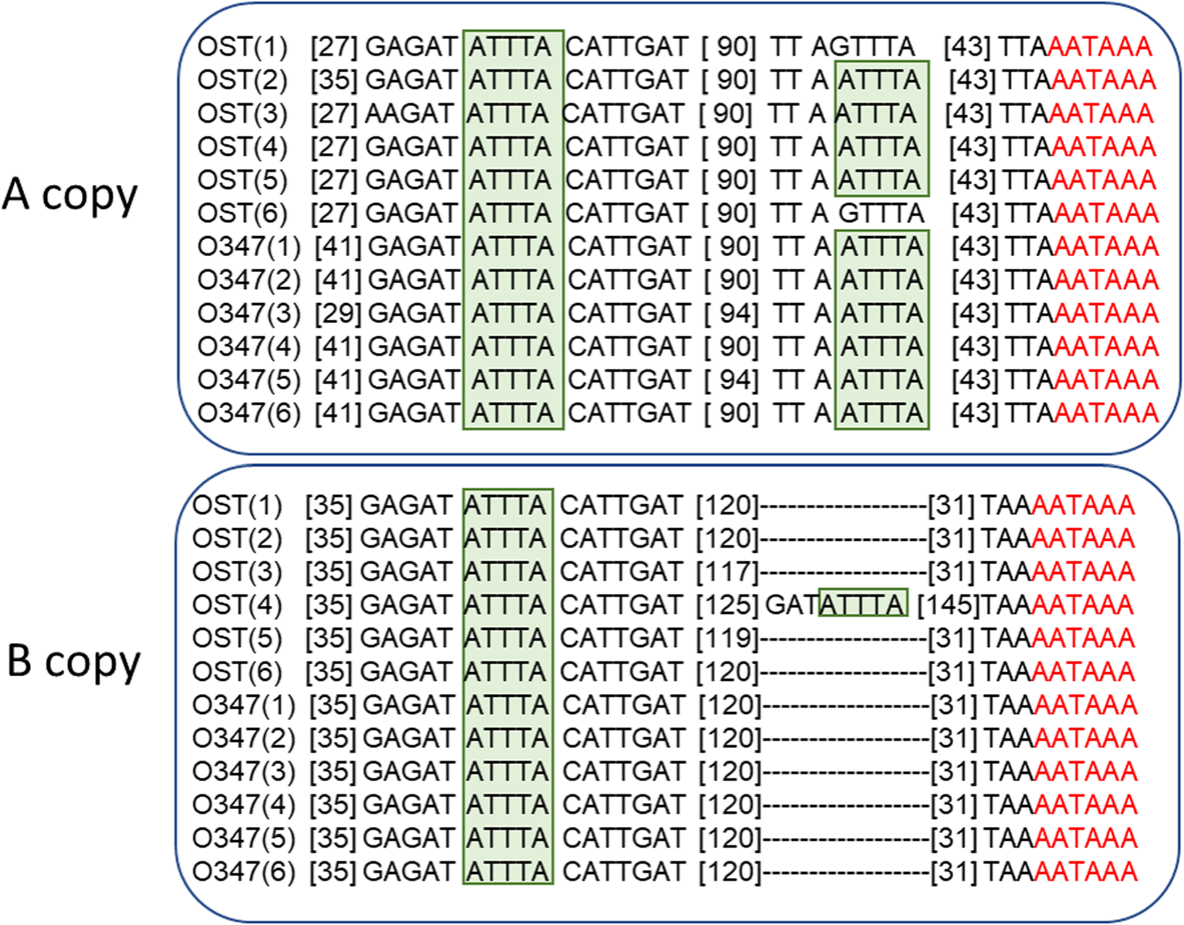
Schematic representation *hsp70* 3′ UTR region of O_ST_ and O_3 + 4 + 7_ lines. The two copies of the gene (A and B) are included. The region represented spans from the stop codon to the polyadenylation signal. Green rectangles: ARE motifs. In red: polyadenylation signal. Square brackets: number of nucleotides from the stop codon and between ARE motifs

The distal regulatory region corresponds to the 3′ untranslated mRNA region (3’UTR), which includes a region spanning 198 ± 4 bp in all *hsp70A* and *hsp70B* copies, except in one *hsp70B* copy from O_ST_ (4*th* line) that is 381 bp long. The in silico screening let us identify conserved regulators consisting in AU-rich elements (AREs), known to be involved in the post-transcriptional regulation of gene expression in *Drosophila* [31] by recruiting ARE-binding proteins that trigger mRNA deadenylation and turnover. In most lines we identified two and one ARE motifs, consisting in a single AUUUA pentamer in *hsp70A* and *hsp70B* copies, respectively. There are a few exceptions; namely, the 1*st* and 6*th* O_ST_ lines that have an unique motif in *hsp70A* (Fig. 2 and Additional file 3) and the 4*th* O_ST_ line, where the 3’UTR region of the *hsp70B* copy is longer and has a second ARE motif (Fig. 2 and Additional file 4). We found that ARE motifs are, in general, highly conserved in both copies of O_ST_ and ${\mathrm{O}}_{\underset{\_}{3+4}+7}$, but the *hsp70A* copy in five ${\mathrm{O}}_{\underset{\_}{3+4}+7}$ lines has a 14 bp additional fragment after the stop codon, which was not found in O_ST_ lines (Additional file 3).

Table 1 summarizes the level of genetic differentiation between chromosomal rearrangements for the complete nucleotide sequence of 5′ and 3′ regulatory regions of A and B copies. Regulatory regions in both copies, except 3’B, exhibited significant genetic differentiation between the two arrangements studied. However, whereas differentiation levels were similar in 5′ regulatory regions for A and B copies, they were significantly higher in 3′ regulatory regions from A copy, where the average number of differences per site (D_xy_) was much higher than in the B copy.

**Table 1:** Genetic differentiation between O_3 + 4 + 7_ and O_ST_ arrangements for 5′ and 3′ regulatory regions

Region	Comparisons	Gen copy	SP_ST_SF_347_	SP_347_SF_ST_	Shared	D_xy_	F_st_	*P*
5’	O_ST_ vs O_3 + 4 + 7_	Conc	17	35	2	0.031	0.344	0.000***
		A (6)	13	33	2	0.034	0.306	0.008**
		B (6)	10	28	2	0.030	0.330	0.045*
3’	O_ST_ vs O_3 + 4 + 7_	Conc	17	38	7	0.127	0.045	0.000***
		A (6)	16	5	0	0.075	0.713	0.007**
		B (6)	1	9	0	0.014	0.081	0.068

Sample sizes are in parenthesis. F_st_: Proportion of nucleotide diversity due to differences between populations; D_xy_: Average number of nucleotide substitutions per site between populations; SP_ST_SF_347_: Sites polymorphic in O_ST_ and fixed in O_3 + 4 + 7_; SP_347_SF_ST_: Sites polymorphic in O_3 + 4 + 7_ and fixed in O_ST_; Conc: concatened data set; Statistical significance was assessed using the *P*-value of Snn statistic; * *P* < 0.05; ** *P* < 0.01; *** *P* < 0.001.

### Basal *hsp70* mRNA expression levels

**Fig. 3:**
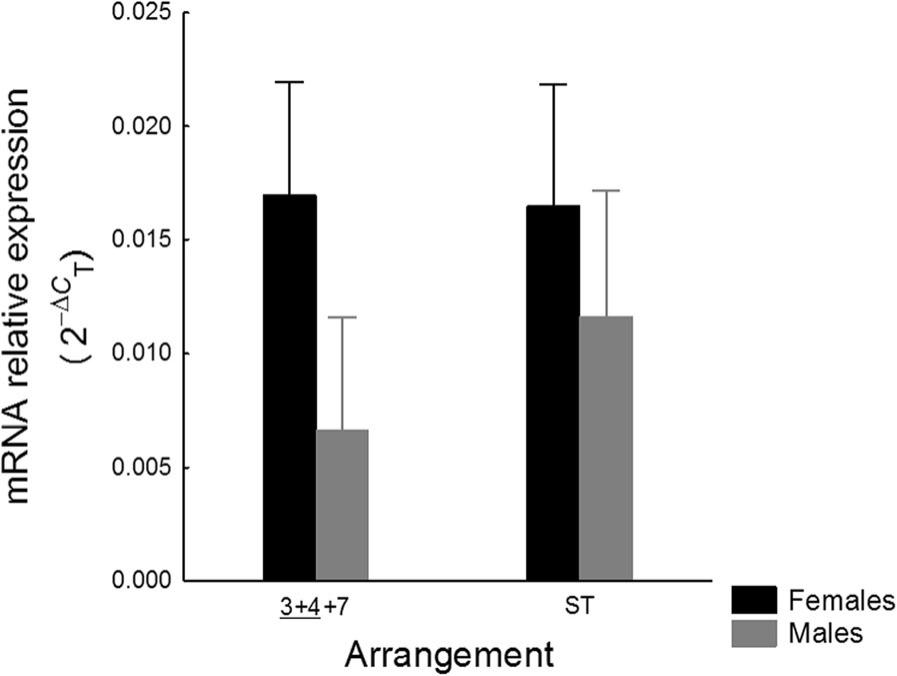
Hsp70 expression in females (black) and males (grey) carrying O_3 + 4 + 7_ and O_ST_ arrangements. Error bars are 95% of confidence intervals

*hsp70* mRNA levels have been quantified in 67 (out of 72) *D. subobscura* samples consisting of 3 biological replicates per isogenic line (6 O_ST_ and 6 ${\mathrm{O}}_{\underset{\_}{3+4}+7}$) and sex, except five lines where only two replicates for females and/or males could be analysed. The ANOVA is shown in Table 2, and results are plotted in Fig. 3. Global *hsp70* mRNA levels were about the same between arrangements (*P* = 0.473) but were higher in females than in males (*P* = 0.005), particularly for the O_3 + 4 + 7_ gene arrangement; but the arrangement × sex interaction was not statistically significant (*P* = 0.296).

**Table 2:** ANOVA for the effects of chromosome gene arrangements (O_3 + 4 + 7_ and O_ST_), isochromosomal line, and sex on mRNA relative expression (${2}^{-\varDelta {C}_{\mathrm{T}}}$). Data plotted in Fig. 3

Source	d.f.	MS	*F*	*P (parametric)*	*P (permutation)*
Arrangement	1	0.000084	0.56	0.473	0.471
Line (Arrangement)	10	0.000152	1.36	0.223	0.222
Sex	1	0.000955	8.55	0.005**	0.004**
Arrangement × Sex	1	0.000125	1.11	0.296	0.294
Error	53	0.000112			

Arrangement was nested within lines. MS: Mean squares, d.f.: degrees of freedom; ** *P* < 0.01.

## Discussion

Here we have sequenced the proximal and distal regulatory regions of the *hsp70* loci and have analysed mRNA expression levels in males and females from 6 isochromosomal lines of warm- (${\mathrm{O}}_{\underset{\_}{3+4}+7}$) and 6 isochromosomal lines of cold-climate (O_ST_) gene arrangements in *D*. *subobscura*. Our aim was to shed some light on the basal amounts of *hsp70* mRNA in these arrangements that show an almost identical genetic organization in the *hsp70* locus [27]. We have now performed for the first time a thorough analysis of 5′ and 3′ *hsp70* regulatory regions together with the quantification of mRNA levels in O_ST_ and O_3 + 4 + 7_ arrangements. We found similar *hsp70* mRNA levels in both arrangements, in accordance with the identical gene configuration found in our previous work [27], but also found differences due to sex (Table 2). Since transcript levels are under the influence of 5′ and 3′ regulatory elements, we analysed these regions and found that the number of 5′ HSE elements in both ${\mathrm{O}}_{\underset{\_}{3+4}+7}$ and O_ST_ arrangements was also identical, as previously observed in other chromosomal arrangements as O_3 + 4 + 8_ and O_3 + 4 + 16_, where these elements evolve in concert through gene conversion [27]. Analysis of 3′ UTR regions also showed no noticeable differences between chromosomal arrangements in the number of ARE elements, considered to be critical regulators of mRNA turnover [31]. However, differences were observed between paralogous genes: in most lines, two ARE motifs were found in *hsp70A* but only one in *hsp70B*, which could point to post-transcriptional regulation events responsible for RNA shortening and degradation. Interestingly, the 3’UTRs were already considered as more divergent between paralogs than the coding and proximal promoter regions [27].

Our main aim here was to correlate the genome organization of *hsp70* loci between cold- (O_ST_) and a warm-climate (${\mathrm{O}}_{\underset{\_}{3+4}+7}$) gene arrangements with mRNA basal expression patterns. The use of isochromosomal lines precludes any extrapolation to wild, outbred flies. mRNA and protein levels are not always correlated [32], and we did not subject our flies to a thermal stress because inbreeding can interfere with the heat-shock response [33]. Besides, inbred individuals might express conditional lethality under heat stress [34]. Therefore, the present results cannot be directly comparable to those obtained by Calabria et al. [14] because these authors measured both basal and heat-induced HSP70 protein levels in outbred strains (obtained from cyclically permuted reciprocal crosses between isochromosomal lines). The differences observed here in the number of ARE motifs among the two *hsp70* copies could contribute to ensure basal amounts of HSP70 protein, basically from one of the gene copies. Moreover, the existence in *D. melanogaster* of 3 and five ARE motifs, respectively, in the two *hsp70* gene clusters compared to *D. subobscura* could contribute to explain why in the previous work [14] certain basal protein amounts were detected. In this regard, a recent study suggested that differences in acclimation capacity between *D. melanogaster* and *D. subobscura* correlate with structural differences in the *hsp70* regulatory regions [35]. However, in view of the highly conserved regulatory elements between chromosomal arrangements found here, we cannot easily explain the differences observed in protein amounts between arrangements [14]. These regulatory elements seem to be particularly highly constrained even though significant genetic differentiation between arrangements was detected when regulatory 5′ and 3′ were considered as a whole (Table 1). This is in concordance with a previous study where differences between arrangements at non-coding regions were also observed [36].

Previous findings pointed out that changes in the expression of particular genes adjacent to inversion breakpoints could contribute to an inversion evolutionary success [37, 38]. However, studies using genome wide transcriptome analyses in *D. melanogaster* [39] and *D. pseudoobscura* [40] concluded that, whereas inversions affect the expression of numerous genes, little evidence of direct position effects was globally detected at the chromosomal level. In *D*. *subobscura* the *hsp70* loci are located in subsection 94A of the hypothetical O_3_ ancestral arrangement. This subsection is inside the derived O_4_ and O_ST_ inversions that gave rise to present day ${\mathrm{O}}_{\underset{\_}{3+4}}$ and O_ST_ gene arrangements, respectively [27]. Using the recently assembled genome of *D*. *subobscura* [41] we have estimated that the *hsp70* loci are around 0.8 Mb from the closest O_4_ distal breakpoint and likely too far away as to be directly affected by this breakpoint, an inference that is in agreement with the similar RNA amounts found in the two chromosomal arrangements analysed here. Four genes (*Pxd*, *Set 8*, *CG5225* and *Acf*) are located near O_4_ inversion breakpoints [42], but at present we do not know whether potential position effects affecting these genes play any role in the in the adaptive value of this inversion. In any case, fitness interactions among loci (epistasis) seem to be important because a recombination load (i.e., the loss of fitness caused by recombination between beneficial combinations of interacting alleles) was detected in ${\mathrm{O}}_{\mathrm{ST}}/{\mathrm{O}}_{\underset{\_}{3+4}+7}$ heterokaryotypes [43].

A potential limitation of our study is that we cannot extrapolate the results to mRNA expression levels during the larval stage. Insects with complex life cycles as *D*. *subobscura* might evolve different thermal sensitivities across life stages, particularly between mobile adults (with a higher capacity for thermoregulatory behaviour) and more sessile (eggs, larvae, pupae) life stages. A previous study assessing the thermal evolution of total mRNA expression levels in *D*. *subobscura* larvae did not detect differential gene expression for *hsp70* among thermal environments, though the highest temperature used in the experiments was likely not high enough as to induce a heat-shock response [44]. We cannot unequivocally generalize our present results to other life stages, but we think that there are not compelling reasons to believe that the basal mRNA expression of *hsp70* would have been different between ${\mathrm{O}}_{\underset{\_}{3+4}}$ and O_ST_ gene arrangements had we used larvae instead of adults.

An important finding is the trend towards higher mRNA amounts in females compared to males. Differences between sexes are, in principle, not surprising because in *Drosophila* a substantial fraction of the transcriptome shows sex-dependent regulation [45]. In our case, the differences could be somehow understandable if the HSP70 protein played sex-specific functions. In *D. melanogaster*, this protein is expressed during spermatogenesis under heat-shock conditions in adults [46], and at normal rearing temperatures in the larval stage and onwards [16, 47]. In the case of females, a novel role for the molecular chaperone HSP70 in the regulation of border cell migration during *Drosophila* oogenesis has also been reported [48].

## Conclusions

The mRNA levels from isogenic lines for O_ST_ and O_3 + 4 + 7_ gene arrangements in *D*. *subobscura* are quantitatively equivalent. This provides some evidence of a parallelism between *hsp70* mRNA expression and its genetic organization. Therefore, the basal levels of *hsp70* mRNA cannot, in principle, explain the spatiotemporal patterns observed for these two gene arrangements. A potential drawback of our study is that we cannot extrapolate the results to wild, outbred flies. Further experiments are necessary to understand the actual role (if any) played by *hsp70* genes in the adaptive value of the rich chromosomal polymorphism on the distal segment of chromosome O, where the *hsp70* genes are located.

## Methods

### Base stocks and fly handling

Wild *D*. *subobscura* males were collected by net-sweeping over banana baits fermented with baker’s yeast in Berbikiz (north of Spain; 43°11′20.31″ N, 3°5′23.74″ W) in 2012. They were individually crossed to three or four virgin females from the *ch*-*cu* marker strain, which carries the recessive markers on the O chromosome *cherry* eyes (*ch*) and *curled* wings (*cu*) and has a highly homogeneous genetic background, to identify the gene arrangements of one set of the five major wild-type chromosomes (the other set of homologous chromosomes coming from the *ch*-*cu* strain). Thereafter, offspring males from each line were crossed to females from the *Va*/*Ba* (*Varicose*/*Bare*) balancer stock [49], which has the same genetic background than the *ch*-*cu* strain, to obtain isochromosomal lines for the O chromosome in an otherwise highly homogeneous genetic background.

A total of 6 O_ST_ and 6 ${\mathrm{O}}_{\underset{\_}{3+4}+7}$ isogenic lines were used in the experiments. All 12 lines were reared at 18 °C (the optimum temperature [50]) with a 12:12 light/dark cycle on David’s killed-yeast *Drosophila* medium [51]. To obtain the experimental flies, eggs from the different lines were placed in 2 × 8 cm vials (45 eggs/vial) with 6 mL of food, which guaranteed low or negligible larval crowding that could have triggered the *hsp70* stress response [20]. For each isogenic line tested, two independent sets of an average of three biological replicates, each containing three 7-day old adult males and females, were used for mRNA analyses.

### Characterization and sequence analysis of the *hsp70* 5′ and 3′ regulatory regions

For each isogenic line, two genomic regions expanding ~ 300 bp upstream the transcription starting site and ~ 205 bp downstream the stop codon were sequenced for the two *hsp70* gene copies (*hsp70B* and *hsp70A*; see [27]). The primers used for amplification of 3′ *hsp70B* and *hsp70A* regulatory regions were, respectively: P154-proximal (5′- ACC CGC AAA ATT GAA CCC AA-3′), CD_1_F (5′-ACC ATA CAG AAC GAC AAG GGT- 3′) and DMT_proximal (5′-AGT CGG AAT TGT GAA GCC TT-3′). Due to the SNPs (Single nucleotide polymorphisms) found in 5’regulatory regions between lines, different primer sets were used: a) *hsp70B*: ARN_1R (5′-TGT GAT GCT TTG GCC CAG AT-3′) and D5_NP (5′-GAT CCT CAA GGA ATG CGA AA-3′)/D7_NP(5′-CTG TAG ATC TTC AGG CAT AAG CTG-3′)/ NP-1-FW (5′ GTA TTG TGG CTT CTT AAC GAG GTTC C-3′); b) *hsp70A:* ARN_1R and and D1_NP (5′-CTT CGG TGG GTT GGA TTG TAG ATT C-3′)/ D3_NP(5′-CTA CTT TCT CTC CTG TGT ATG TCT GG-3′)/D4_NP(5′-CTC TCC TGT GTA CAT ATG TCT TGC G-3′). To identify several CREs (Cis-Regulatory Elements) that are typically found in the proximal 5′ flanking region, the sequences of the two *hsp70* genes were first separately aligned using the MAFFT E-INS-i algorithm [52] available at: https://mafft.cbrc.jp/alignment/server/. Next, we used the information available on the *hsp70* proximal promoters of *D. subobscura* [27] and other *Drosophila* species [29] to identify the conserved heat shock elements in the sequences of the 12 isogenic lines (HSEs 1–4), and the Eukaryotic Neural Network Promoter Prediction software (http://www.fruitfly.org/seq_tools/promoter.html) to find other CREs such as GAGA binding sites and some core promoter elements, including the transcription start site (TSS) and the TATA box. 3′ UTR sequences were aligned using the MAFFT E-INS-i algorithm [52] and manually refined using the Genedoc alignment editor (version 2.7) [53]. The web-based ARE score application (http://arescore.dkfz.de/arescore.pl) was used to identify post-transcriptional regulators of mRNA levels such as AU-rich elements [54]. The DNASp version v6.12 program [55] was used for genetic differentiation analyses between arrangements, assessed using D_xy_ [56] and F_st_ [57], and the significance for Snn [58] was obtained with 1000 replicates. Analyses were performed excluding gaps.

### Quantification of *hsp70* transcripts by qRTPCR

Total RNA extraction was performed separately in adult males and females using the Trizol Lysis reagent (Thermo Fisher Scientific Inc.) following manufacturer’s instructions, and then treated with 1 μl (2 U/μl) of Ambion DNase I (Thermo Fisher Scientific Inc.) 4 h at 37 °C. Concentration of DNA-free samples was checked in a Nanodrop-2000 spectrophotometer (Thermo Scientific Nanodrop) and adjusted to ~ 1 μg/μL and then cDNA synthesis was carried out with anchored-Oligo (dT)18 primers using the Transcriptor First Strand cDNA Synthesis kit (Roche Diagnostics SL, SPAIN). Gene expression was quantified by measuring fluorescence intensity using iQ SYBR Green Supermix (BioRad, Hercules CA, USA) on a CFX96 BioRad Real-Time lightcycler at 59 °C using *hsp70* specific primers. All assays were performed in three technical replicates, and the *rp49* housekeeping gene was used as an endogenous control because this gene showed to be expressed equally in all samples analysed (*P* = 0.772, see Additional file 5). Relative expression of *hsp70* was then calculated with the comparative *C*_T_ method [59] and *ΔC*_T_ values for the set of samples are provided in Additional file 6.

For each isogenic line, we amplified a conserved *hsp70* coding fragment of 226 bp, common to both *hsp70* copies described in *D*. *subobscura* [27]. The primers used for amplification were: HSP70_1L (5′-CAC AGT CTT TGA CGC CAA GC-3′) and HSP70_1R (5′-TGT GAT GCT TTG GCC CAG AT-3′). For the *rp49* housekeeping gene, the primers RP49F (5′-ACA TCG GTT ATG GCT CCA C-3′) and RP49R (5′-GAT TTC CTT GCG CTT CTT TG-3′) were designed from a *D. subobscura* GenBank sequence (Accession: AJ228921), amplifying a 164 bp segment from the gene’s second exon. Primer efficiencies of *hsp70* and *rp49*, calculated on a standard curve slope using serially diluted templates, were 98 and 99.6%, respectively.

### Statistical methods

*Hsp70* mRNA expression levels were analysed using the linear model.

*y*_*ijkl*_ = *μ* + *A*_*i*_ + *L*_*j*(*i*)_ + *S*_*k*_ + *AS*_*ik*_ + *ε*_*ijkl*_, (1)

where *μ* is the overall grand mean, *A*_*i*_ is the fixed effect of gene arrangement (O_ST_, ${\mathrm{O}}_{\underset{\_}{3+4}+7}$), *L*_*j*(*i*)_ is the random effect of the *j*th isochromosomal line (*j* = 1, 2, ⋯, 6) within arrangement *i*, *S*_*k*_ is the fixed effect of sex, and *ε*_*ijkl*_ is the residual error associated with the mRNA expression levels of the *l*th replicate from the *k*th sex with the *i*th gene arrangement from the *j*th isochromosomal line. Following Schmittgen and Livak [59], statistical tests for mRNA relative expression were performed after the ${2}^{-\varDelta {C}_{\mathrm{T}}}$ transformation.

To avoid potential problems with the standard assumption that errors are independent, and identically distributed as normal random variables with common variance and zero expectation, we also performed permutation tests [60, 61] to check the validity of parametric *P*-values. In model (1) the permutation test is a four-stage process. First, random permutation among lines, within arrangements, for the among-line *F*_line(arrangement)_; second, random permutation among lines and arrangements for the between-arrangements *F*_arrangement_; third, random permutation between sexes for the between-sex *F*_sex_; and fourth, random permutation between arrangements and sexes for the arrangement × sex interaction *F*_arrangement × sex_. In each case, 9999 random allocations plus the observed *F*-values were obtained, which gave us 10,000 values of each *F*-statistics for each dependent variable. We then calculate the percentage of replications under the null hypothesis that provide as large a value of each *F* as the obtained *F*.

The equal expression of *rp49* gene was tested on Ct values using one-way ANOVA with four sample groups: females O_ST_, males O_ST_, females ${\mathrm{O}}_{\underset{\_}{3+4}+7}$ and males ${\mathrm{O}}_{\underset{\_}{3+4}+7}$_._ The Levene’s test was used to test the homogeneity of variances between groups.

Analyses were performed using the statistical software STATISTICA 8.0 [62]. Permutation tests were implemented in the algebra environment MATLAB using tools supplied by the Statistics Toolbox [63].

## Supplementary information

**Additional file 1. **Multiple sequence alignment of 5’proximal promoters of *hsp70*A.

**Additional file 2. **Multiple sequence alignment of 5’proximal promoters of *hsp70*B.

**Additional file 3. **Multiple sequence alignment of *hsp70*A 3’UTR.

**Additional file 4. **Multiple sequence alignment of *hsp70*B 3’UTR.

**Additional file 5. **Statistical results for the homogeneous expression of *rp49* housekeeping gene.

**Additional file 6.** Raw gene expression data.

## Data Availability

The data analysed in this study are included in this article and its additional files. All newly reported sequences in this study were deposited in GenBank/EMBL/DDBJ database under accession numbers: MK862365- MK862404.

## Abbreviations

ARE: AU-rich element
CREs: Cis-regulatory elements
HSEs: Heat shock elements
HSF: Heat shock factor
SNPs: Single nucleotide polymorphisms
UTR: Untranslated region
