# Supplementary material for: Basal hsp70 expression levels do not explain adaptive variation of the warm- and cold-climate O3 + 4 + 7 and OST gene arrangements of Drosophila subobscura
Source: BMC Evol Biol. 2020 Jan 31;20:17. doi: 10.1186/s12862-020-1584-z (PMC6995229; doi:10.1186/s12862-020-1584-z)
Supplement: Supplementary file 1 — Additional file 1. Multiple sequence alignment of 5’proximal promoters of hsp70A. [file 12862_2020_1584_MOESM1_ESM.pdf]

Additional file 1: Identification of several conserved CREs in the 5' *cis*-regulatory region of the Hsp70A gene in 12 *D. subobscura* lines isogenic for the O_{ST} and the $O_{\underline{3+4}+7}$ gene arrangements. The sequences of two of the lines below have been described in Puig-Giribets et al. (2018) and are available in Genbank under the following accession numbers: OST (1): MG780233, OST (2): MG780234. Green boxes refer to four conserved HSEs described in Puig-Giribets et al. (2018) in the Hsp70 proximal promoters of *D. subobscura*. The three central nucleotides of each nGAAn/nTTCn unit have been colored in yellow. Nucleotides in red correspond to polymorphic sites. Pink boxes represent conserved GAGA sites in blue (G-: CTCTC; G+: GAGAG). The TATA box and the transcription start site (TSS) sequences have been underlined.

```
                  HSE4
OST (1)       ATCGAATTTTCTCGATTCCCAAATAAAACGGTTTTTTGCGGTAGGTCAAGTACATTG
OST (2)       ATCGAATTTTCTCGATTCCCAAATAAAAC-GTTTTTTGCGGTAGGTCAAGTACATTG
OST (3)       ATCGAATTTTCTCGATTCCCAAATAAAACGGTTTTTTGCGGTAGGTCAAGTACATTG
OST (4)       ATCGAATTTTCTCGATTCCCAAATAAAACGTTTTTTTGCGGTAGGTCAAGTACATTG
OST (5)       ATCGAATTTTCTCGATTCCCAAATAAAAAGGTTTTTTGCGGTAGGTCAAGTACATTG
OST (6)       ATCGAATTTTCTCGATTCCCAAATAAAACGTTTTTTTGCGGTAGGTCAAGTACATTG
O3+4    (1)   ATCGAATTTTCTCGATTCCCAAATAAAACGGTTTTTTGCGGTAGGTCAAGTACATTG
O3+4    (2)   ATCGAATTTTCTCGATTCCCAAATAAAACGGTTTTTTGCGGTAGGTCAAGTACATTG
O3+4    (3)   ATCGAAATTTCTGGATTTCCAAATAAAACGGCTTGTTGCGGTAGGTCAAGTACATTG
O3+4    (4)   ATCGAATTTTCTCGATTCCCAAATAAAACGGTTTTTTGAGGTAGGTCAAGTACATTG
O3+4    (5)   ATCGAATTTTCTCGATTCCCAAATAAAAC-GTTTTTTGCGGTAGGTCAAGTACATTG
O3+4    (6)   ATCGAAATTTCTGGATTTCCAAATAAAACGGCTTGTTGCGGTAGGTCAAGTACATTG
              ****** ***** **** ***********   ** *** ******************

                      HSE3                       G-
OST (1)       GCACAGAGAAAAGTCGAGAAATTTCGTCAACAAATCACCCTCTCTT-CCAACACAAGCTG
OST (2)       GCACAGAGAAAAGTCGAGAAATTTCGTCAACAAATCACCCTCTCTT-CCAACACAAGCTG
OST (3)       GCACAGAGAAAAGTCGAGAAATTTCGTCAACAAATCACCCTCTCTT-CCAACACAAGCTG
OST (4)       GCACAGAGAAAAGTCGAGAAATTTCGTCAACAAATCACCCTCTCTT-CCAACACAAGCTG
OST (5)       GCACAGAGAAAAGTCGAGAAATTTCGTCAACAAATCACCCTCTCTT-CCAACACAAGCTG
OST (6)       GCACAGAGAAAAGTCGAGAAATTTCGTCAACAAATCACCCTCTCTT-CCAACACAAGCTG
O3+4    (1)   GCACAGAGAAAAGTCGAGAAATTTCGTCAACAAATCACCCTCTCTT-CCAATACAAGCTG
O3+4    (2)   GCACAGAGAAAAGTCGAGAAATTTCGTCAACAAATCACCCTCTCTT-CCAATACAAGCTG
O3+4    (3)   GCACAGAGAAAAATCGAGAAATTTCGTTCACAATTCACCCTCTCTTACAAACACAAGCCC
O3+4    (4)   GCACAGAGAAAAGTCGAGAAATTTCGTCCAAAATTTACCCTCTCTTACAAACACAAGCCC
O3+4    (5)   GCACAGAGAAAAGTCGAGAAATTTCGTCAACAA-TCACCCTCTCTT-CCAACACAAGCTG
O3+4    (6)   GCACAGAGAAAAATCGAGAAATTTCGTTCACAATTCACCCTCTCTTACAAACACAAGCCC
              ************ **************  * ** * ********** * ** ******
```

```
                     G-
OST  (1)      CTTGCGTTCTCTCT-------GCTGCCAGCTGCCAAGACTTGTTGTCTCGCTCTGACGCA
OST  (2)      CTTGCGTTCTCTCT-------GCTGCCAGCTGCCAAGACTTGTTGTCTCGCTCTGACGCA
OST  (3)      CTTGCGTTCTCTCT-------GCTGCCAGCTGCCAAGACTTGTTGTCTCGCTCTGACGCA
OST  (4)      CTTGCGTTCTCTCTGCTGCCAGCTGCCAGCTGCCAAGACTTGTTGTCTCGCTCTGACGCA
OST  (5)      CTTGCGTTCTCTCT-------GCTGCCAGCTGCCAAGACTTGTTGTCTCGCTCTGACGCA
OST  (6)      CTTGCGTTCTCTCTGCTGCCAGCTGCCAGCTGCCAAGACTTGTTGTCTCGCTCTGACGCA
O3+4   (1)    CTTGCGTTCTCTCT-------GCTGCCAGCTGCCAAGACTTGTTGTCTCGCTCTGACGCA
O3+4   (2)    CTTGCGTTCTCTCT-------GCTGCCAGCTGCCAAGACTTGTTGTCTCGCTCTGACGCA
O3+4   (3)    ATTGTGCTCTCTCT-------GCTGCCAGCTGCCAAGACTTGTCGTCTCGCTCTGACGCA
O3+4   (4)    ATTGTGCTCTCTTT-------GCTGCCAGCTGCCAAGACTTGTCGTCTCGCTCTGACGCA
O3+4   (5)    CATGCGTTCTCTCT-------GCTGCCAGCTGCCAAGACTTGTCGTCTCGCTCTGACGCA
O3+4   (6)    ATTGTGCTCTCTCT-------GCTGCCAGCTGCCAAGACTTGTCGTCTCGCTCTGACGCA
                ** * ***** *       ********************** ****************

                                HSE2       G+     HSE1
OST  (1)      TGCGATTTAGTGAAACATTCAAGATATTTCTAGAAGAGAGCTCTCGAAGTTTCGCAGCCA
OST  (2)      TGCGATTCAGGGTAACGTTCAAGATATTTCTAGAAGAGAGCTCTCGAAGTTTCGCAGCCA
OST  (3)      TGCGATTTAGTGTAACATTCAAGATATTTCTAGAAGAGAGCTCTCGAAGTTTCGCAGCCA
OST  (4)      TGCGATTCAGGGTAACGTTCAAGATATTTCTAGAAGAGAGCTCTCGAAGTTTCGCAGCCA
OST  (5)      TGCGATTCAGGGTAACGTTCAAGATATTTCTAGAAGAGAGCTCTCGAAGTTTCGCAGCCA
OST  (6)      TGCGATTCAGGGTAACGTTCAAGATATTTCTGGAAGAGAGCTCTCGAAGTTTCGCAGCCA
O3+4   (1)    TGCGATTTAGTGTAACATTCAAGATATTTCTAGAAGAGAGCTCTCGAAGTTTCGCAGCCA
O3+4   (2)    TGCGATTTAGTGTAACATTCAAGATATTTCTAGAAGAGAGCTCTCGAAGTTTCGCAGCCA
O3+4   (3)    TGCGATTCAGGGTAACATTCAAGATATTTCTAGAAGAGAGCTCTCGAAGTTTCGCAGCCT
O3+4   (4)    TGCGATTCAGGGTAACATTCAAGATATTTCTAGAAGAGAGCTCTCGAAGTTTCGCAGCCT
O3+4   (5)    TGCGATTCAGGGTAACATTCAAGATATTTCTAGAAGAGAGCTCTCGAAGTTTCGCAGCCT
O3+4   (6)    TGCGATTCAGGGTAACATTCAAGATATTTCTAGAAGAGAGCTCTCGAAGTTTCGCAGCCT
              *******.** * ***.**************.***************************

                           TATA BOX (-32)          TRANSCRIPTION START SITE (TSS) (+1)
OST  (1)      GAGCGGCCGGGTATAAATACAGCCGACAGTTTCTCTTCGCAGCAATTCAAACCAAACAAG
OST  (2)      GAGCGGCCGGGTATAAATACAGCCGACAGTTTCTCTTCGCAGCAATTCAAACCAAACAAG
OST  (3)      GAGCGGCCGGGTATAAATACAGCCGACAGTTTCTCTTCGCAGCAATTCAAACCAAACAAG
OST  (4)      GAGCGGCCGGGTATAAATACAGCCAACAGTTTCTCTTTGCAGCAATTCAAACCAAACAAG
OST  (5)      GAGCGGCCGGGTATAAATACAGCCAACAGTTTCTCTTCGCAGCAATTCAAACCAAACAAG
OST  (6)      GAGCGGCCGGGTATAAATACAGCCAACAGTTTCTCTTTGCAGCAATTCAAACCAAACAAG
O3+4   (1)    GAGCGGCCGGGTATAAATACAGCCGACAGTTTCTCTTCGCAGCAATTCAAACCAAACAAG
O3+4   (2)    GAGCGGCCGGGTATAAATACAGCCGACAGTTTCTCTTCGCAGCAATTCAAACCAAACAAG
```

```
O3+4   (3)   GAGCGGCCGGGTATAAATACAGCCGACAGTTTCTCTTCTCAGCAATTCAAACCAAACAAG

O3+4   (4)   GAGCGGCCGGGTATAAATACAGCCGACAGTTTCTCTTCTCAGCAATTCAAACCAAACAAG

O3+4   (5)   GAGCGGCCGGGTATAAATACAGCCGACAGTTTCTCTTCTCAGCAATTCAAACCAAACAAG

O3+4   (6)   GAGCGGCCGGGTATAAATACAGCCGACAGTTTCTCTTCTCAGCAATTCAAACCAAACAAG

             ************************ ************  *********************
```
