# Supplementary material for: Basal hsp70 expression levels do not explain adaptive variation of the warm- and cold-climate O3 + 4 + 7 and OST gene arrangements of Drosophila subobscura
Source: BMC Evol Biol. 2020 Jan 31;20:17. doi: 10.1186/s12862-020-1584-z (PMC6995229; doi:10.1186/s12862-020-1584-z)
Supplement: Supplementary file 2 — Additional file 2. Multiple sequence alignment of 5’proximal promoters of hsp70B. [file 12862_2020_1584_MOESM2_ESM.pdf]

Additional file 2: Identification of several conserved CREs in the 5' *cis*-regulatory region of the Hsp70B gene in 12 *D. subobscura* isogenic lines for the O_{ST} and the O_{3+4+7} gene arrangements. Some lines shown in the alignment below have been described in Puig-Giribets et al. (2018) and are available in Genbank under the following accession numbers: OST (1): MG780233, OST (2): MG780234. Green boxes refer to four conserved HSEs described in Giribets et al. (2018) in the Hsp70 proximal promoters of *D. subobscura*. The three central nucleotides of each nGAAn/nTTCn unit have been colored in yellow. Nucleotides in red correspond to polymorphic sites. Pink boxes represent conserved GAGA sites (G-: CTCTC; G+: GAGAG) in blue. The TATA box and the transcription start site (TSS) sequences have been underlined.

```
                     HSE4

OST (1)      atagaattttctcgattcccaaataaaacggttttttgcggtaggtcaagtacatt
OST (2)      atcgaattttctcgattcccaaataaaac-gtttttgcggtaggtcaagtacatt
OST (3)      atcgaattttctcgattcccaaataaaacggttttttgcggtaggtcaagtacatt
OST (4)      atcgaattttctcgattcccaaataaaac-gtttttgcggtaggtcaagtacatt
OST (5)      atcgaattttctcgattcccaaataaaacgtttttttgcggtaggtcaagtacatt
OST (6)      atcgaattttctcgattcccaaataaaac-gtttttgcggtaggtcaagtacatt
O3+4    (1)  atcgaattttctcgattcccaaataaaacggttttttgcggtaggtcaagtacatt
O3+4    (2)  atcgaattttctcgattcccaaataaaac-gtttttgcggtaggtcaagtacatt
O3+4    (3)  atcgaattttctcgattcccaaataaaacgggtttttgaggtaggtcaagtacatt
O3+4    (4)  atcgaattttctcgattcccaaataaaacggttttttgaggtaggtcaagtacatt
O3+4    (5)  atcgaattttctcgattcccaaataaaacgggtttttgaggtaggtcaagtacatt
O3+4    (6)  atcgaattttctcgattcccaaataaaacggttttttgaggtaggtcaagtacatt
             ** ****************************    ****** *****************
```

```
                          HSE3                   G-

OST (1)      ggcacagagaaaagtcgagaaatttcgtcaacaaatcaccctctctt-ccaacacaagct
OST (2)      ggcacagagaaaagtcgagaaatttcgtcaacaaatcaccctctctt-ccaacacaagct
OST (3)      ggcacagagaaaagtcgagaaatttcgtcaaaaaatcaccctctctt-ccaacacaagct
OST (4)      ggcacagagaaaagtcgagaaatttcgtcaacaaatcaccctctctt-ccaacacaagct
OST (5)      ggcacagagaaaagtccagaaatttcgtcaacaaatcaccctctctt-ccaacacaagct
OST (6)      ggcacagagaaaagtcgagaaatttcgtcaacaaatcaccctctctt-ccaacacaagct
O3+4    (1)  ggcacagagaaaagtcgagaaatttcgtcaacaaatcaccctctctt-ccaatacaagct
O3+4    (2)  ggcacagagaaaagtcgagaaatttcctcaacaaatcaccctctctt-ccaacacaagct
O3+4    (3)  ggcacagagaaaagtcgagaaatttcgtccacaatttacactctcttacaaacacaagcc
O3+4    (4)  ggcacagagaaaagtcgagaaatttcgtccacaatttaccctctcttacaaacacaagcc
O3+4    (5)  ggcacagagaaaagtcgagaaatttcgtcaacaa-tcaccctctctt-ccaacacaagct
O3+4    (6)  ggcacagagaaaagtcgagaaatttcgtccacaattcaccctctcttacaaacacaagcc
             **************** ********* ** * ** *.** ******* * **.******.
```

```
                          G-
OST  (1)          gcttgcgttctctct-------gctgccagctgccaagacttgttgtctcgctctgacgc
OST  (2)          gcttgcgttctctct-------gctgccagctgccaagacttgttgtctcgctctgacgc
OST  (3)          gcttgcgttctctct-------gctgccagctgccaagacttgttgtctcgctctgacgc
OST  (4)          gcttgcgttctctctgctgccagctgccagctgccaagacttattgtctcgctctgacgc
OST  (5)          gcttgcgttctctct-------gctgccagctgccaagacttgttgtctcgctctgacgc
OST  (6)          gcttgcgttctctctgctgccagctgccagctgccaagacttgttgtctcgctctgacgc
O3+4    (1)       gcttgcgttctctct-------gctgccagctgccaagacttgttgtctcgctctgacgc
O3+4    (2)       gcatgcgttctctct-------gctgccagctgccaagacttgttgtctcgctctgacgc
O3+4    (3)       cattgtgctctctct-------gctgccagctgccaagacttgtcgtctcgctctgacgc
O3+4    (4)       cattgtgctctcttt-------gctgccagctgccaagacttgtcgtctcgctctgacgc
O3+4    (5)       gcatgcgttctctct-------gctgccagctgccaagacttgtcgtctcgctctgacgc
O3+4    (6)       cattgtgctctcttt-------gctgccagctgccaagacttgtcgtctcgctctgacgc
                     ** * ***** *       ******************** * ***************

                                             HSE2       G+      HSE1
OST  (1)          atgcgatttagtgtaacattcaagatatttctagaagagagctctcgaagtttcgcagccag
OST  (2)          atgcgattcagggtaacgttcaagatatttctagaagagagctctcgaagtttcgcagccag
OST  (3)          atgcgatttagtgtaacattcaagatatttctagaagagagctctcgaagtttcgcagccag
OST  (4)          atgcgatttagtgtaacattcaagatatttctagaagagagctctcgaagtttcgcagccag
OST  (5)          atgcgattcagggtaacgttcaagatatttctagaagagagctctcgaagtttcgcagccag
OST  (6)          atgcgattcagggtaacgttcaagatatttctagaagagagctctcgaagtttcgcagccag
O3+4    (1)       atgcgatttagtgtaacattcaagatatttctagaagagagctctcgaagtttcgcagccag
O3+4    (2)       atgcgatttagtgtaacattcaagatatttctagaagagagctctcgaagtttcgcagccag
O3+4    (3)       atgcgattcagggtaacattcaagatatttctagaagagagctctcgaagtttcgcagcctg
O3+4    (4)       atgcgattcagggtaacattcaagatatttctagaagagagctctcgaagtttcgcagcctg
O3+4    (5)       atgcgattcagggtaacattcaagatatttctagaagagagctctcgaagtttcgcagcctg
O3+4    (6)       atgcgattcagggtaacattcaagatatttctagaagagagctctcgaagtttcgcagcctg
                  ******** ** ***** ****************************************** *

                      TATA BOX (-32)    TRANSCRIPTION START SITE (TSS) (+1)
OST  (1)          agcggccgggtataaatacagccgacagtttctcttcgcagcaattca
OST  (2)          agcggccgggtataaatacagccgacagtttctcttcgcagcaattca
OST  (3)          agcggccgggtataaatacagccgacagtttctcttcgcagcaattca
OST  (4)          agcggccgggtataaatacagccgacagtttctcttcgcagcaattca
OST  (5)          agcggccgggtataaatacagccaacagtttctcttcgcagcaattca
OST  (6)          agcggccgggtataaatacagccaacagtttctcttcgcagcaattca
O3+4    (1)       agcggccgggtataaatacagccgacagtttctcttcgcagcaattca
O3+4    (2)       agcggccgggtataaatacagccgacagtttctcttcgcagcaattca
O3+4    (3)       agcggccgggtataaatacagccgacagtttctcttctcagcaattca
```

O3+4	(4)	agcggccgggtataaatacagccgacagtttctcttctcagcaattca
O3+4	(5)	agcggccgggtataaatacagccgacagtttctcttctcagcaattca
O3+4	(6)	agcggccgggtataaatacagccgacagtttctcttctcagcaattca
		************************************* **********
