# Supplementary material for: Basal hsp70 expression levels do not explain adaptive variation of the warm- and cold-climate O3 + 4 + 7 and OST gene arrangements of Drosophila subobscura
Source: BMC Evol Biol. 2020 Jan 31;20:17. doi: 10.1186/s12862-020-1584-z (PMC6995229; doi:10.1186/s12862-020-1584-z)
Supplement: Supplementary file 3 — Additional file 3. Multiple sequence alignment of hsp70A 3’UTR. [file 12862_2020_1584_MOESM3_ESM.pdf]

Additional file 3: Alignment of hsp70A 3'UTR in 12 isogenic lines from *D. subobscura*.

ARE sites are underlined and polyadenylation sites represented in red.

```
OST(1)_hsp70A     GCACACTC--------------ATCAGACCCCGTAGCCATAGAGATATTTACATTGATGA
OST(2)_hsp70A     GCGCATTCCACAT------CACATCCACATCCATAGCCATAGAGATATTTACATTGATGG
OST(3)_hsp70A     GCACACTC--------------ATCAGACCCCGTAGCCATAAAGATATTTACATTGATGA
OST(4)_hsp70A     GCACACTC--------------ATCAGACCCCGTAGCCATAGAGATATTTACATTGATGG
OST(5)_hsp70A     GCACACTC--------------ATCAGACCCCGTAGCCATAGAGATATTTACATTGATGG
OST(6)_hsp70A     GCACACTC--------------ATCAGACCCCGTAGCCATAGAGATATTTACATTGATGA
O347(1)_hsp70A    GCGCATTCCACATCACATCCACATCCACATCCATAGCCATAGAGATATTTACATTGATGG
O347(2)_hsp70A    GCGCATTCCACATCACATCCACATCCACATCCATAGCCATAGAGATATTTACATTGATGG
O347(3)_hsp70A    GCGCATTCCACAT------------CACATCCATAGCCATAGAGATATTTACATTGATGG
O347(4)_hsp70A    GCGCATTCCACATCACATCCACATCCACATCCATAGCCATAGAGATATTTACATTGATGG
O347(5)_hsp70A    GCGCATTCCACATCACATCCACATCCACATCCATAGCCATAGAGATATTTACATTGATGG
O347(6)_hsp70A    GCGCATTCCACATCACATCCACATCCACATCCATAGCCATAGAGATATTTACATTGATGG
                  ** ** **                      ** ******** *****************

OST(1)_hsp70A     ATTATTTCACAGTTCA----TCTTATGTTTAAAAAAGTGATAAGGATGTTTGGTTGATCT
OST(2)_hsp70A     ATTATTTCATAGTTCA----TTTTATGTTTAAAAAAGTGATAAGGATTTTTGGTTGATCT
OST(3)_hsp70A     ATTATTTCACAGTTCA----TTTTATGTTTAAAAAAGTGATAAGGATGTTTGGTTGATCT
OST(4)_hsp70A     ATTATTTCATAGTTCA----TTTTATGTTTAAAAAAGTGATAAGGATGTTTGGTTGATCT
OST(5)_hsp70A     ATTATTTCACAGTTCA----TTTTATGTTTAAAAAAGTGATAAGGATGTTTGGTTGATCT
OST(6)_hsp70A     ATTATTTCACAGTTCA----TCTTATGTTTAAAAAAGTGATAAGGATGTTTGGTTGATCT
O347(1)_hsp70A    ATTATTTCATAGTTCA----TTTTATGTTTAAAAAAGTGATAAGGATGTTTGGTTGATCT
O347(2)_hsp70A    ATTATTTCATAGTTCA----TTTTATGTTTAAAAAAGTGATAAGGATGTTTGGTTGATCT
O347(3)_hsp70A    ATTATTTCATAGTTCATTTTTTTTATGTTTAAAAAAGTGATAAGGATGTTTGGTTGATCT
O347(4)_hsp70A    ATTATTTCATAGTTCA----TTTTATGTTTAAAAAAGTGATAAGGATGTTTGGTTGATCT
O347(5)_hsp70A    ATTATTTCATAGTTCATTTTTTTTATGTTTAAAAAAGTGATAAGGATGTTTGGTTGATCT
O347(6)_hsp70A    ATTATTTCATAGTTCA----TTTTATGTTTAAAAAAGTGATAAGGATGTTTGGTTGATCT
                  ********* ******    * ************************* ************

OST(1)_hsp70A     CTCCACAAACTTTCCTGGTATTAGCGACTGAGTTAGTTTAGTTTTATGTTAGCAACCAAT
OST(2)_hsp70A     CTCCACAAACTTTCCTGGTATTAGCGACTGAGTTAATTTAGTTTTATGTTAGCAACAAAT
OST(3)_hsp70A     CTCCACAAACTTTCCTGGTATTAGCGACTGAGTTAATTTAGTTTTATGTTAGCAGCCAAT
OST(4)_hsp70A     CTCCACAAACTTTCCTGGTATTAGCGACTGAGTTAATTTAGTTTTATGTTAGCAACCAAT
OST(5)_hsp70A     CTCCACAAACTTTCCTGGTATTAGCGACTGAGTTAATTTAGTTTTATGTTAGCAACCAAT
OST(6)_hsp70A     CTCCACAAACTTTCCTGGTATTAGCGACTGAGTTAGTTTAGTTTTATGTTAGCAACCAAT
O347(1)_hsp70A    CTCCACAAACTTTCCTAGTATTAGCGACTGAGTTAATTTAGTTTTATGTTAGCAACCAAT
O347(2)_hsp70A    CTCCACAAACATTCCTAGTATTAGCGACTGAGTTAATTTAGTTTTATGTTAGCAACCAAT
O347(3)_hsp70A    CTCCACAAACTTTCCTAGTATTAGCAGCTGAGTTAATTTAGTTTTATGTTAGCAACCAAT
O347(4)_hsp70A    CTCCACAAACATTCCTAGTATTAGCGACTGAGTTAATTTAGTTTTATGTTAGCAACCAAT
O347(5)_hsp70A    CTCCACAAACTTTCCTAGTATTAGCGACTGAGTTAATTTAGTTTTATGTTAGCAACCAAT
O347(6)_hsp70A    CTCCACAAACATTCCTAGTATTAGCGACTGAGTTAATTTAGTTTTATGTTAGCAACCAAT
                  ********** ***** ********  ******** ****************** * ***

OST(1)_hsp70A     TGTAATTGCTTAAGTTAGAAGTCTTAAATAAA
OST(2)_hsp70A     TGTAATTGCTTAAGTTAGAAGTCTTAAATAAA
OST(3)_hsp70A     TGTAATTGCTTAAGTTAGAAGTCTTAAATAAA
OST(4)_hsp70A     TGTAATTGCTTAAGTTAGAAGTCTTAAATAAA
OST(5)_hsp70A     TGTAATTGCTTAAGTTAGAAGTCTTAAATAAA
OST(6)_hsp70A     TGTAATTGCTTAAGTTAGAAGTCTTAAATAAA
O347(1)_hsp70A    TGTTATTCTTTAAGTTAGAAGTCTTAAATAAA
O347(2)_hsp70A    TGTTACTCTTTAAGTTAGAAGTCTTAAATAAA
O347(3)_hsp70A    TGTAATTCTTTAAGTTAGAAGTCTTAAATAAA
O347(4)_hsp70A    TGTTACTCTTTAAGTTAGAAGTCTTAAATAAA
O347(5)_hsp70A    TGTTACTCTTTAAGTTAGAAGTCTTAAATAAA
O347(6)_hsp70A    TGTTACTCTTTAAGTTAGAAGTCTTAAATAAA
                  *** * *  ***********************
```
