# Supplementary material for: Basal hsp70 expression levels do not explain adaptive variation of the warm- and cold-climate O3 + 4 + 7 and OST gene arrangements of Drosophila subobscura
Source: BMC Evol Biol. 2020 Jan 31;20:17. doi: 10.1186/s12862-020-1584-z (PMC6995229; doi:10.1186/s12862-020-1584-z)
Supplement: Supplementary file 4 — Additional file 4. Multiple sequence alignment of hsp70B 3’UTR. [file 12862_2020_1584_MOESM4_ESM.pdf]

Additional file 4: Alignment of hsp70B 3'UTR in 12 isogenic lines from *D. subobscura* .

ARE sites are underlined and polyadenylation signal in red

```
OST(1)_hsp70B   GCGCATTCCACATCACATCCACATCCATAGCCATAGAGATATTTACATTGATGGATTATT
OST(2)_hsp70B   GCGCATTCCACATCACATCCACATCCATAGCCATAGAGATATTTACATTGATGGATTATT
OST(3)_hsp70B   GCGCATTCCACATCACATCCACATCCATAGCCATAGAGATATTTACATTGATGGATTATT
OST(4)_hsp70B   GCGCATTCCACATCACATCCACATCCATAGCCATAGAGATATTTACATTGATGGATTATT
OST(5)_hsp70B   GCGCATTCCACATCACATCCACATCCATAGCCATAGAGATATTTACATTGATGGATTATT
OST(6)_hsp70B   GCGCATTCCACATCACATCCACATCCATAGCCATAGAGATATTTACATTGATGGATTATT
O347(1)_hsp70B  GCGCATTCCACATCACATCCACATCCATAGCCATAGAGATATTTACATTGATGGATTATT
O347(2)_hsp70B  GCGCATTCCACATCACATCCACATCCATAGCCATAGAGATATTTACATTGATGGATTATT
O347(3)_hsp70B  GCGCATTCCACATCACATCCACATCCATAGCCATAGAGATATTTACATTGATGGATTATT
O347(4)_hsp70B  GCGCATTCCACATCACATCCACATCCATAGCCATAGAGATATTTACATTGATGGATTATT
O347(5)_hsp70B  GCGCATTCCACATCACATCCACATCCATAGCCATAGAGATATTTACATTGATGGATTATT
O347(6)_hsp70B  GCGCATTCCACATCACATCCACATCCATAGCCATAGAGATATTTACATTGATGGATTATT
                ************************************************************

OST(1)_hsp70B   TCATAGTTCATTTTATGTTTGACTGATAAGAATGTTTAGCTGTGTTTGGTATTAGAATGT
OST(2)_hsp70B   TCATAGTTCATTTTATGTTTGACTGATAAGAATGTTTAGCTGTGTTTGGTATTAGAATGT
OST(3)_hsp70B   TCATAGTTCATTTTATGTTTGACTGATAAGAATGTTTAGCTGTGTTTGGTATTAGAATGT
OST(4)_hsp70B   TCATAGTTCATTTTATGTTTGACTGATAAGAATGTTTAGCTGTGTTTGGTATTATTTTTT
OST(5)_hsp70B   TCATAGTTCATTTTATGTTTGACTGATAAGAATGTTTAGCTGTGTTTGGTATTAGAATGT
OST(6)_hsp70B   TCATAGTTCATTTTATGTTTGACTGATAAGAATGTTTAGCTGTGTTTGGTATTAGAATGT
O347(1)_hsp70B  TCATGGTTCATTTTATGTTTGACTGATAAGAATGTTTAGCTGTGTTTGGTATTAGAATGT
O347(2)_hsp70B  TCATAGTTCATTTTATGTTTGACTGATAAGAATGTTTAGCTGTGTTTGGTATTAGAATGT
O347(3)_hsp70B  TCATAGTTCATTTTATGTTTGACTGATAAGAATGTTTAGCGAAGATTGGTATTAGAATGT
O347(4)_hsp70B  TCATGGTTCATTTTATGTTTGACTGATAAGAATGTTTAGCTGTGTTTGGTATTAGAATGT
O347(5)_hsp70B  TCATAGTTCATTTTATGTTTGACTGATAAGAATGTTTAGCTGTGTTTGGTATTAGAATGT
O347(6)_hsp70B  TCATAGTTCATTTTATGTTTGACTGACAAGAATGTTTAGCGAAGATTGGTATTAGAATGT
                **** ********************* *************   * *********   * *

OST(1)_hsp70B   TT-----AGTATTAGCGCCTCGAATTATTCTAGTTTTATTTTAGCAACAAATTTTAA---
OST(2)_hsp70B   TT-----AGTATTAGCGCCTCGAATTATTCTAGTTTTATTTTAGCAACAAATTTTAA---
OST(3)_hsp70B   TT-----AGTATTAGCGCCTCGA---ATTCTAGTTTTATTTTAGCAGCAAATTTTAA---
OST(4)_hsp70B   TTTTTTTGATATTTAATCGCTACAGCGAAATTGGTTGGTCGCAAAAATGAGTCCGCATTG
OST(5)_hsp70B   TT-----AGTATT-GCGCCTCGAATTATTCTAGTTTTATTTTAGCAACAAATTTTAA---
OST(6)_hsp70B   TT-----AGTATTAGCGCCTCGAATTATTCTAGTTTTATTTTAGCAACAAATTTTAA---
O347(1)_hsp70B  TT-----AGTATTAGCGCCTCGAATTATTCTAGTTTTATTTTAGCAACAAATTTTAA---
O347(2)_hsp70B  TT-----AGTATTAGCGCCTCGAATTATTCTAGTTGTATTTTAGCAACAAATTTTAA---
O347(3)_hsp70B  TT-----AGTATTAGCGCCTCGAATTATTCTAGTTTTATTTTAGCAACAAATTTTAA---
O347(4)_hsp70B  TT-----AGTATTAGCGCCTCGAATTATTCTAGTTTTATTTTAGCAACAAATTTTAA---
O347(5)_hsp70B  TT-----AGTATTAGCGCCTCGAATTATTCTAGTTTTATTTTAGCAACAAATTTTAA---
O347(6)_hsp70B  TT-----AGTATTAGCGCCTCGAATTATTCTAGTTTTATTTTAGCAACAAATTTTAA---
                **       ****    *            * * *   *   *  *   * *    *

OST(1)_hsp70B   ------------------------------------------------------------
OST(2)_hsp70B   ------------------------------------------------------------
OST(3)_hsp70B   ------------------------------------------------------------
OST(4)_hsp70B   ACCAGGTACTTCAGTTTTGTGTTGAATATTATAAAAAGTTACTGAAAATTTTGAACTCAA
OST(5)_hsp70B   ------------------------------------------------------------
OST(6)_hsp70B   ------------------------------------------------------------
O347(1)_hsp70B  ------------------------------------------------------------
O347(2)_hsp70B  ------------------------------------------------------------
O347(3)_hsp70B  ------------------------------------------------------------
O347(4)_hsp70B  ------------------------------------------------------------
O347(5)_hsp70B  ------------------------------------------------------------
O347(6)_hsp70B  ------------------------------------------------------------

OST(1)_hsp70B   ----------------------------------------ATTCCTAAGTTAGTGAAAGG
OST(2)_hsp70B   ----------------------------------------ATTCCTAAGTTAGTGAAAGG
OST(3)_hsp70B   ----------------------------------------ATTCCTAAGTTAGTGAAAGG
OST(4)_hsp70B   AAAAGAGTTGTTTGCCGAACGAACACTAGAATAGAATATGATTCCTAAGTTAGTGAAAGG
OST(5)_hsp70B   ----------------------------------------ATTCCTAAGTTAGTGAAAGG
OST(6)_hsp70B   ----------------------------------------ATTCCTAAGTTAGTGAAAGG
O347(1)_hsp70B  ----------------------------------------ATTCCTAAGTTAGTGAAAGG
O347(2)_hsp70B  ----------------------------------------ATTCCTAAGCTAGTGAAAGG
O347(3)_hsp70B  ----------------------------------------ATTCCTAAGTTAGTGAAAGG
O347(4)_hsp70B  ----------------------------------------ATTCCTAAGTTAGTGAAAGG
O347(5)_hsp70B  ----------------------------------------ATTCCTAAGTTAGTGTAAAG
O347(6)_hsp70B  ----------------------------------------ATTCCTAAGTTAGTGAAAGG
                                                        ********* ***** ** *

OST(1)_hsp70B   TCTAAAATAAA
OST(2)_hsp70B   TCTAAAATAAA
OST(3)_hsp70B   TCTAAAATAAA
OST(4)_hsp70B   TCTAAAATAAA
OST(5)_hsp70B   TCTAAAATAAA
OST(6)_hsp70B   TCTAAAATAAA
O347(1)_hsp70B  TCTAAAATAAA
O347(2)_hsp70B  TCTAAAATAAA
O347(3)_hsp70B  TCTAAAATAAA
O347(4)_hsp70B  TCTAAAATAAA
O347(5)_hsp70B  TCTAAAATAAA
O347(6)_hsp70B  TCTAAAATAAA
                ***********
```
