# Supplementary material for: Basal hsp70 expression levels do not explain adaptive variation of the warm- and cold-climate O3 + 4 + 7 and OST gene arrangements of Drosophila subobscura
Source: BMC Evol Biol. 2020 Jan 31;20:17. doi: 10.1186/s12862-020-1584-z (PMC6995229; doi:10.1186/s12862-020-1584-z)
Supplement: Supplementary file 5 — Additional file 5. Statistical results for the homogeneous expression of rp49 housekeeping gene. [file 12862_2020_1584_MOESM5_ESM.pdf]

Additional file 5: Test for the equal expression and homogeneity of variances of *rp49* gene between groups

TEST	Source	d.f.	SS	*F*	*P*
**ANOVA**	Between groups	3	0.658	0.375	0.772

				Levene's satistic	*P*
**LEVENE**		3		0.869	0.463

Groups considered: Females O_{ST}, males O_{ST}, females O_{3+4+7}, males O_{3+4+7}
